# Dormancy activation mechanism of tracheal stem cells

**DOI:** 10.18632/oncotarget.8179

**Published:** 2016-03-18

**Authors:** Xin Li, Jing-xian Xu, Xin-Shan Jia, Wen-ya Li, Yi-chen Han, En-hua Wang, Fang Li

**Affiliations:** ^1^ Department of Physiology, College of Life Science and Biopharmaceutics of Shenyang Pharmaceutical University, Shenyang, China; ^2^ Department of Pathology, College of Basic Medical Sciences, China Medical University, Shenyang, China; ^3^ Department of Pathology, First Affiliated Hospital of China Medical University, Shenyang, China; ^4^ Department of Ophthalmology, The 4th Affiliated Hospital, Eye Institute, China Medical University, The Key Laboratory of Lens Research, Shenyang, China; ^5^ Department of Thoracic Surgery, The First Affiliated Hospital, China Medical University, Shenyang, China; ^6^ IVF Michigan, Bloomfield Hills, MI, USA

**Keywords:** methylation, stem cell, dormancy, Sox2 expression

## Abstract

Accurate markers and molecular mechanisms of stem cell dormancy and activation are poorly understood. In this study, the anti-cancer drug, 5-fluorouracil, was used to selectively kill proliferating cells of human bronchial epithelial (HBE) cell line. This method can enrich and purify stem cell population. The dormant versus active status of stem cells was determined by phosphorylation of RNAp II Ser2. The surviving stem cells were cultured to form stem cell spheres expressing stem cell markers and transplanted into nude mice to form a teratoma. The results demonstrated the properties of stem cells and potential for multi-directional differentiation. Bisulfite sequencing polymerase chain reaction showed that demethylation of the Sox2 promoter by 5-FU resulted in Sox2 expression in the dormant stem cells. This study shows that the dormancy and activation of HBE stem cells is closely related to epigenetic modification.

## INTRODUCTION

Adult stem cells in the body are generally in a state of dormancy or the G0 phase of the cell cycle. Stem cells can be activated to re-enter the cell cycle via stimulation by specific environmental or internal factors [1–3]. However, the underlying mechanism is relatively unclear. At present, studies on stem cell activation and dormancy are mainly focused on hematopoietic [4–7], melanocyte [2], epidermal [8, 9], and cancer stem cells [10–12]. Several theories have been proposed to explain the dormancy and activation mechanism of stem cells, including phosphorylation of RNA polymerase [13, 14], p27 gene regulation [15, 16], regulation of TGF-β/Smad pathway [17], cell dormancy based on autophagy, biochronometer theory, and insulin/Igf1 pathway. It is known that 5-FU concentrates stem cells and increases the proportion of cells in the G0 phase [18, 19, 20, 21]. We induced tracheal epithelium cells into tracheal epithelium stem cells using 5-FU in serum-free culture, which formed teratomas after transplantation into nude mice. The results of this study demonstrated a new method of inducing stem cells without gene transduction. This study also explored the relationship between the activation and dormancy of tracheal stem-like cells and epigenetic molecular mechanisms.

## RESULTS

### Treatment with 5-FU inhibits proliferation of HBE cells

We used MTT method to analyze HBE cell proliferation. The proliferation rate of serum-free cultured cell spheres was significantly higher than untreated HBE cell. The proliferation rate of 5- FU treated HBE cells was the slowest. Based on the results, median inhibitory concentration (IC50) of 5-FU (40 μg/mL) for 24 h was chosen as the dose used for subsequent experiments [20].

Cell cycle distribution was assayed with 5-FU treated cells and serum-free cultured cells. G0 phase population increased which contains stem cells. Both methods can enrich stem cells.

**Figure 1:**
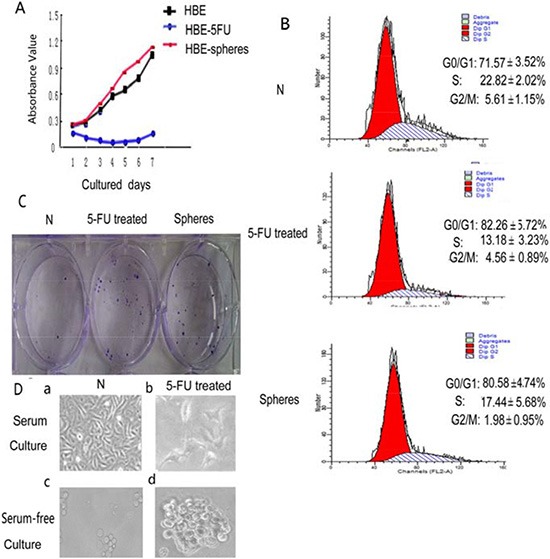
The comparison between HBE cells, 5-FU-treated cells and serum-free cultured spheres (**A**) The proliferation of HBE cells, 5-FU-treated HBE cells and serum-free cultured HBE cells were assessed using the MTT assay. The proliferation rate of 5- FU treated HBE cells was the slowest. (*p* < 0.05) (**B**) Effect of 5-FU treatment and serum-free culture on HBE cell cycle distribution. Cell cycle distribution of G0/G1, S, and G2/M phases was measured by DNA/PI flow cytometry. Values are expressed as mean ± SD. (**C**) Clone-forming assay using untreated HBE cells, 5-FU-treated HBE cells and serum-free cultured HBE cells. (**D**) Cell morphology of untreated HBE cells, 5-FU treated HBE cells, serum-free cultured HBE cell spheres and serum-free cultured 5-FU-treated HBE cell spheres.

After treatment with 5-FU, the HBE cell population in S phase decreased from 22.82 ± 2.02% to 13.18 ± 3.23%, whereas the cell population in G1/G0 phase increased from 71.57 ± 3.52% to 82.26 ± 5.72%. Thus, 5-FU treatment caused cell cycle arrest in the G1/G0 phase, with only slight effects on cells in other phases of the cell cycle. Since most stem cells are in G0 phase, we hypothesized that 5-FU treatment might result in the enrichment of stem cells. Whereas serum-free cultured HBE cell sphere population in S phase decreased from 22.82 ± 2.02% to 17.44 ± 5.68%, whereas the cell population in G1/G0 phase increased from 71.57 ± 3.52% to 80.58 ± 4.74%. All the comparisons above are significant (Figure 1B).

### Both 5-FU treated cells and serum-free cultured cells exhibit high clonogenic capacities

Only 7.0 ± 1.06% of HBE cells were able to form clones. 5-FU-treated HBE cells was 24.5 ± 4.63% (Figure 1C). Statistical analysis revealed significant differences in clone formation efficiency between 5-FU treated and untreated cell populations (*p* < 0.01). The clone-forming capacity of serum-free cultured HBE cell spheres was 28.0 ± 3.78%, serum-free cultured HBE cell spheres were able to form 4 times clones than untreated HBE cells (*p* < 0.01; Figure 1C).

### HBE cells that survive 5-FU treatment exhibit a high capacity for sphere formation

The vast majority of HBE cells died after 24 hrs treated with 5-FU (Figure 1Db); however, a small proportion of the HBE cells survived and generated floating spherical colonies after 10 days in culture (Figure 1Dd). Survived HBE cells after 5-FU treatment exhibited a higher capacity for sphere formation (Figure 1Dd). The spheres of 5-FU-treated cells grew faster and larger (Figure 1Dd) than those untreated HBE cells (Figure 1Dc).

### Both 5-FU treatment and serum-free culture induced demethylation of Sox2, and activated stem cells

**Figure 2:**
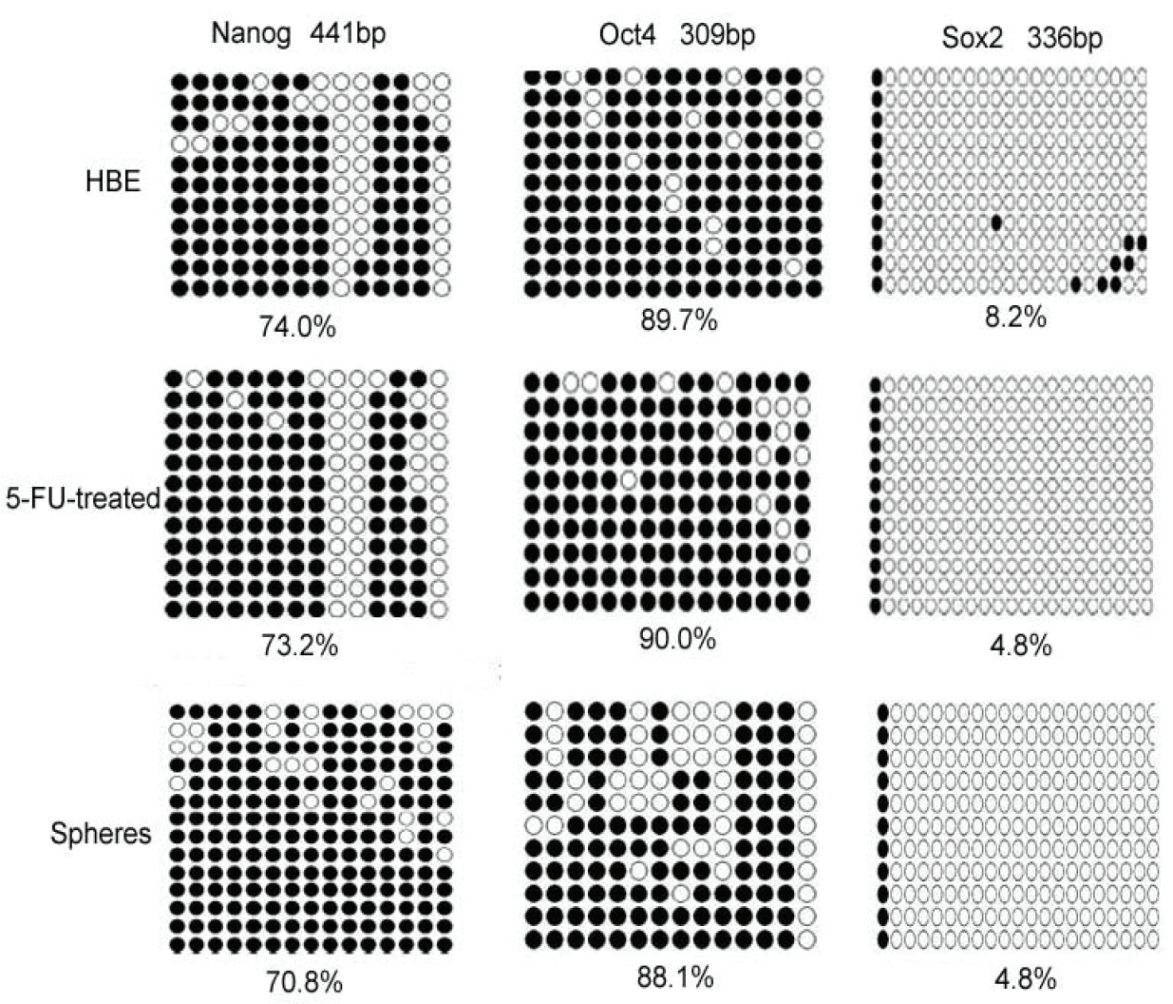
The methylation status of HBE cells, 5-FU treated cells and serum-free cultured cells Both treatment of HBE cells with 5-FU and culturing in serum-free medium decreased the methylation of the stem cell transcription factors Sox2 remarkably. Open circle, unmethylation of the gene promoter; closed circle, methylation of the gene promoter.

Control cells (untreated) showed 89.7% methylation of Oct4, 74.0% methylation of Nanog, and 8.2% methylation of Sox2. In contrast, 5-FU-treated group showed 90.0% methylation level of Oct4, 73.2% methylation of Nanog. Compared with control group, the methylation of Oct4 and Nanog changed weakly. The methylation of the Sox2 promoter remarkably decreased from 8.2% to 4.8%, leading to its activation (Figure 2).

Serum-free cultured group showed 88.1% methylation level of Oct4, 70.8% methylation of Nanog. Compared with HBE group, the methylation of Oct4 and Nanog changed weakly. The methylation of the Sox2 promoter decreased from 8.2% to 4.8%, leading to its activation (Figure 2).

Both 5-FU-treated group and serum-free cultured group showed 4.8% methylation level of Sox2, whereas control HBE cells showed 8.2% methylation level of Sox2. Both methods activated stem cells.

### 5-FU treated and serum-free cultured HBE cells promote formation of teratomas after transplantation

**Figure 3:**
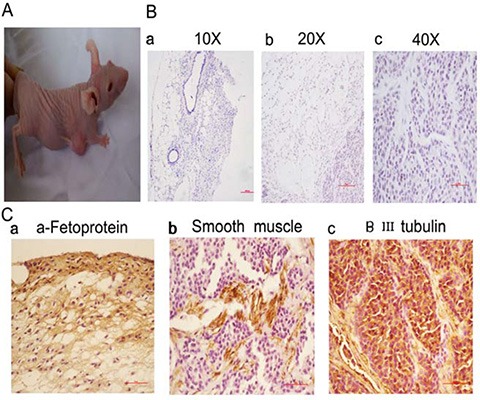
Treatment of HBE cells with 5-FU and culturing in serum-free medium leads to teratomas *in vivo* (**A**) Mice inoculated with 3 × 10^5^ serum-free cultured 5-FU-treated HBE cells were euthanized after 5 weeks. (**B**) HE staining results of the tumors (a, 10 ×; b, 20 ×; c, 40 ×, Scale bar, 100 μm). (**C**) Immunohistochemical staining results of the tumors (40 ×, Scale bar, 50 μm). a. The expression of the endodermal marker α-Fetoprotein. b. The expression of the mesodermal marker Smooth muscle c. The expression of the ectodermal marker βIII tubulin.

To assess the tumor forming potential, 3 × 10^5^ HBE cells and 3 × 10^5^ serum-free cultured 5-FU-treated HBE cells were injected into mice and tumor formation was monitored. Five weeks after injection, all three mice injected with serum-free cultured 5-FU-treated HBE cells had tumors with an average volume of 600 mm^3^ (Figure 3A), whereas no tumor growth was observed after inoculation with untreated HBE cells.

Pathology results confirmed that the tumors formed by serum-free cultured 5-FU-treated HBE cells were teratomas. Teratoma showed differentiation into cell types from three germ layers. HE staining showed the tissues containing squamous epithelia (ectoderm), glandular epithelia (endoderm) and smooth muscle (mesoderm) (Figure 3B). The immunohistochemical staining showing expression of the lineage markers α-Fetoprotein (endoderm) (Figure 3Ca), Smooth muscle actin (mesoderm) (Figure 3Cb) and βIII tubulin (ectoderm) (Figure 3Cc) from three independent derivations. Teratoma formation demonstrated the pluripotency.

### 5-FU enhances the expression of stem cell markers in HBE cells

**Figure 4:**
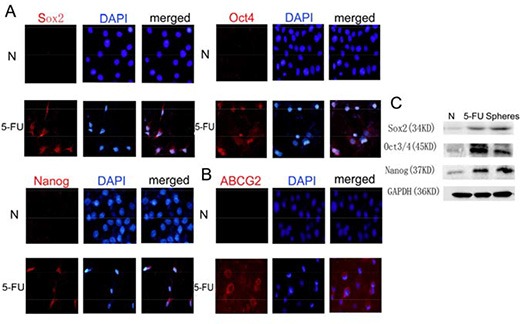
Treatment with 5-FU can enrich the stem cell population in HBE cells (**A**) Immunofluorescence staining of Sox2, Oct4 and Nanog in HBE cells with or without 5-FU treatment. In untreated HBE cells, few Sox2, Oct4 or Nanog-positive cells were observed. After 5-FU treatment, the number of Sox2, Oct4 or Nanog-positive cells increased remarkably. Nuclei were counterstained with DAPI (blue). (**B**) Immunofluorescence staining of ABCG2 in HBE cells with or without 5-FU treatment. (**C**) Changes in the expression of Sox2, Oct4 and Nanog proteins in untreated HBE cells, 5-FU-treated HBE cells and serum-free cultured HBE cells.

There was little Oct4, Nanog, or Sox2 expression in the parental HBE cells. However, after treatment with 5-FU at the IC50 of 40 μg/mL for 24 h, the expression of Oct4 (Figure 4Ab), Nanog (Figure 4Ac), and especially Sox2 (Figure 4Aa), increased, and ABCG2 also became positive (Figure 4B). The Western blot data showed trends for Oct4, Sox2 and Nanog that were similar to those observed by immunofluorescence (Figure 4C).

### Differences of dormant tracheal stem cells, activated tracheal stem cells and terminally differentiated tracheal cells

**Figure 5:**
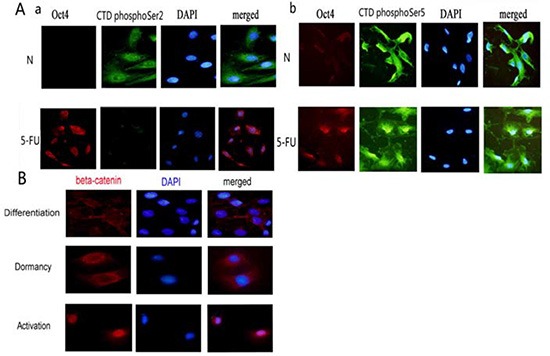
Differences of dormant tracheal stem cells, activated tracheal stem cells and terminally differentiated tracheal cells (**A**)The expressions of RNA polymerase II CTD phospho-Ser2 or RNA polymerase II CTD phospho-Ser5 in Oct4 negative HBE cells and Oct4 positive HBE cells. a. The relationship of RNA polymerase II CTD phospho-Ser2 and Oct4. b. The relationship of RNA polymerase II CTD phospho-Ser5 and Oct4. (**B**)The expressions of β-catenin in differentiated HBE cells, in dormant HBE cells and in activated HBE cells.

To clarify the differences between 5-FU-treated and untreated HBE cells, we co-stained cells with antibodies against Oct4 and RNA polymerase II CTD phospho-Ser2 or RNA polymerase II CTD Phospho-Ser5 and performed fluorescence microscopy. Oct4 was negative in untreated differentiated HBE cells, and RNA polymerase II CTD phospho-Ser2 was positive. Oct4 was positive in 5-FU-treated HBE cells, and RNA polymerase II CTD phospho-Ser2 was negative (Figure 5Aa). At this time, cells stayed in the resting states of stem cells. Oct4 was negative and RNA polymerase II CTD phospho-Ser5 was positive in untreated differentiated HBE cells. Oct4 was positive in 5-FU treated HBE cells, and CTD polyIISer5 was positive, too (Figure 5Ab). RNA polymerase II CTD phospho-Ser5 was not related to the states of stem cells. In untreated HBE cells, β-catenin was present at the cell membrane. In contrast, after 5-FU treatment, cells exhibited substantially higher levels of total and nuclearβ-catenin compared with untreated HBE cells (Figure 5B). Thus, high levels of nuclear accumulation of β-catenin are consistent with activation of stem cells.

## DISCUSSION

### Characterization and identification of dormant tracheal stem cells

Dormant tracheal stem cells are generally in G0 phase, but not all G0 cells are stem cells; some are differentiated. The difference between stem cells and differentiated cells in G0 phase is that the former may re-enter the cell cycle, while the latter will exit the cell cycle and perish. A decline of mRNA synthesis causes cells to enter the dormant period and thus regulates cell cycle and metabolism. The initiation and maintenance of mRNA transcription and subsequent RNA polymerase II release by DNA are closely related to carboxy-terminal domain phosphorylation of RNA polymerase II. Most differentiated and senescent cells actively synthesize mRNA. In these cells, RNA polymerase II of Ser2 and Ser5 is phosphorylated irrespectively of the cell cycle.

Phosphorylation of RNA polymerase II is absent in dormant cells (G0 phase) such as T lymphocytes. However, RNA polymerase II phosphorylation status of tracheal dormant stem cells is unknown. We treated tracheal cells with 5-FU and cultured them in serum-free medium, thus enriching stem cells in G0 phase. Stem cells can be isolated from a mixed culture containing differentiated cells. Studies have shown that cells with negative expression of RNA polymerase II Ser2 phosphorylation and positive expression of perinuclear Oct4 or Sox2 are dormant stem cells. RNA polymerase II Ser5 phosphorylation is not related to the states of stem cells. Since the activation of stem cells is associated with Wnt pathways, the location of β-catenin expression can be used to indicate the status of the cells, where nuclear expression would indicate activation and cytoplasmic expression would indicate differentiation [22, 23, 24].

Some scholars have suggested that dormant stem cells are spherical, whereas differentiated cells have multiple apophyses. We believe that cell shape is an inadequate indicator of dormant or active status.

### 5-FU causes Sox2 DNA promoter demethylation and reactivation of tracheal stem cells

In this study, HBE cells were treated with 5-FU, resulting in a decrease in numbers of cells in the S phase and thus, cells in G0/G1 phase were concentrated. This phenomenon has been previously observed in several tumor cells from the gastrointestinal tract [25, 26], lung [27–34], and liver [35]. Treatment of HBE cells with 5-FU resulted in decrease in methylation of Sox2 DNA promoter from 8.4% to 4.8%, and the methylation island disappeared. The process involving demethylation of DNA promoter and activation of Sox2 gene is very similar to that observed in embryonic stem cell activation and dormancy [36–44]. DNA methylation correlates closely to gene silencing. Unmethylated DNA regions are associated with gene activation.

### 5-FU treatment in serum-free medium induced HBE cells to form tracheal stem cells, whose successful purification was confirmed by teratoma formation after transplantation into nude mice

HBE cells treated with 5-FU in serum-free culture assumed a spherical shape instead of an adherent culture. Thereafter, these cells formed a teratoma when transplanted into nude mice. The tumor was positive for ectodermal marker, β III-tubulin; mesodermal marker Smooth muscle; and endodermal marker α-Fetoprotein. HBE cells can develop into pseudostratified ciliated columnar cells in serum and 3-dimensional culture, and purified stem cells can be obtained after treatment. Further, stem cells can be purified from cancer cell lines, including those of breast cancer, lung cancer, and oral cancer. This study showed that adult stem cells could be obtained from 5-FU and serum-free culture *in vitro*.

## CONCLUSIONS

1. Cells negative for RNA polymerase II and positive for perinuclear Oct4 or Sox2 are dormant stem cells. That can be used to identify dormant stem cells.
2. 5-FU can cause Sox2 DNA promoter demethylation, which in turn reactivates tracheal stem cells from dormancy to the active state.
3. Teratomas formed after transplantation of serum-free cultured 5-FU-treated HBE cells, which confirmed successful purification of tracheal stem cells. Thus, the results of this study demonstrated a new method to induce stem cells without altering cellular genetics.

## METHODS

### Cell culture and treatment

The human bronchial epithelial cell line HBE was obtained from American Type Culture Collection. HBE cells were cultured in RPMI 1640 (Gibco, USA) supplemented with 10% fetal bovine serum (FBS), 100 units/mL penicillin (Sigma), and 100 μg/mL streptomycin (Sigma). Cells were grown on sterilized culture dishes and were passaged every 2 days with 0.25% trypsin (Invitrogen).

### MTT assay

HBE variability was assessed using the MTT assay. Approximately 5 × 10^3^ cells/well were seeded into 96-well culture plates and incubated under normal culture conditions for 12, 24, 48 and 72 h. The cells were then incubated with 20 μL MTT (10 mg/mL) for 4 h at 37°C, and 200 μL DMSO was added to solubilize the formazan product for 20 min at room temperature. The optical density (OD) was determined using a spectrophotometer (Bio-800, Bio-Rad, USA) at a wavelength of 570 nm.

### Cell cycle analysis

The cell cycle was assayed by measuring DNA fragment staining with propidium iodide (PI). In brief, HBE cells, 5-FU treated cells and sphere cells were harvested and washed twice with phosphate-buffered saline (PBS) and fixed in ice-cold 70% ethanol at 4°C overnight. Ethanol-fixed cells were centrifuged and washed once with PBS. The cell pellet was then suspended in 0.1 mL RNaseA (5 mg/mL) and incubated in a 37°C shaker for 30 min. Then, the cells were stained with a propidium iodide (50 μg/mL) and incubated in the dark for 30 min at 37°C. In total, 10, 000 cells were harvested. The cells were analyzed immediately by exciting PI at 488 nm and measuring the emission at 580 nm using a BD canto II flow cytometer (BD Biosciences, San Jose, CA, USA) with Cell Quest 3.0 software.

### Western blotting

HBE cells were incubated on ice for 20 min in 50 μL of RIPA buffer supplemented with 1 mM PMSF, 1 μg/mL leupeptin, 1 mM β-glycerophosphate, 2.5 mM sodium pyrophosphate, and 1 mM Na3VO4. Following centrifugation at 12, 000 × g for 20 min at 4°C, the supernatant was transferred to a new EP tube for determination of the concentration of the protein content. Next, 60μg protein from each sample was resolved on 10% sodium dodecyl sulfate (SDS) polyacrylamide gels and transferred to polyvinylidene fluoride (PVDF) membranes. Membranes were then blocked in TBST solution containing 4% skim milk for 2 h at room temperature. After washing, the membrane was incubated overnight at 4°C with specific primary antibodies at appropriate concentration in 1% BSA solution. After washing the membrane three times for 15 min with TBST solution, the membrane was further incubated with appropriate HRP-conjugated secondary antibodies in TBST solution for 2 h at room temperature. The membrane was washed three times for 15 min by TBST solution and incubated with ECL solution for 1 min. Protein bands were visualized using the ECL chemiluminescence method. Relative protein levels were quantified using GAPDH as loading control.

### Cell immunofluorescence

Cells were fixed in 4% paraformaldehyde in 20 mM HEPES (pH 7.4) for 20 min, washed three times, and permeabilized with 1.0% Triton X-100 for 5 min. Cells were then incubated with rabbit polyclonal anti-Sox2 antibody, rabbit polyclonal anti-Oct4 antibody, rabbit RNA Polymerase II CTD Repeat Antibody (Phospho-Ser2) antibody, rabbit RNA Polymerase II CTD Repeat Antibody (Phospho-Ser5) antibody, rabbit polyclonal anti-β-catenin antibody, and mouse polyclonal anti-Nanog antibody for 1 h at room temperature before being washed three times and incubated with goat anti-rabbit conjugated secondary antibody and goat anti-mouse conjugated secondary antibody for 30 min at room temperature in the dark. DAPI was used for nuclear counterstaining. The stained cells were mounted and viewed under a BX51 inverted epifluorescence microscope (Olympus, Tokyo, Japan).

### Culturing of cell spheres

Cells were resuspended in serum-free DMEM-F12 medium supplemented with 20 ng/mL EGF (BD Biosciences), 20 ng/mL bFGF (BD Biosciences), 1 × B27 supplement (Invitrogen), 1 × N2 supplement (Invitrogen), and 4 μg/mL insulin (Sigma-Aldrich) and plated at a density of 10000 cells/mL and plated at 500 μL per well in ultra-low attachment 24-well plates (Corning, NY, USA). The medium was replaced or supplemented with fresh growth factors twice per week.

### Analysis of tumorigenic properties of HBE, 5-FU treated cells and spheres

This study was performed with the approval of the Care and Use of Laboratory Animals Committee of China Medical University, Shenyang, China, and all of the experiments were performed according to the National Institutes of Health Guide for the Care and Use of Laboratory Animals. Male BALB/C nude mice (aged 6–7 weeks) were divided into HBE, serum-free cultured 5-FU-treated HBE cell spheres (*n* = 3 per group) and received 3 × 10^5^ cells by intraperitoneal injection (i.p.) at the lower left quadrant before they were euthanized at 5 weeks after transplantation. The resulting tumors were measured using a Vernier caliper, weighed, and photographed. Tumor samples were removed and fixed in 10% formaldehyde, and were embedded in paraffin for subsequent hematoxylin and eosin (HE) and immunohistochemical staining to assess tumor pathology.

### Immunohistochemistry

Nude mice tumor specimens were fixed with 10% neutral formalin and embedded in paraffin, and 4-μm-thick sections were prepared. Immunostaining was performed using the avidin–biotin–peroxidase complex method (Ultrasensitive™, MaiXin, Fuzhou, China). Paraffin sections were dewaxed in xylene and rehydrated in graded alcohols. Antigen retrieval was performed by heating the sections for 1.5 min in 0.01 mol/L citrate buffer, pH 6.0. Non-specific staining was reduced by incubation in blocking buffer containing goat serum (SP KIT-B1; Maixin-Bio, Fuzhou, China) for 30 min. Then, the sections were incubated with α-Fetoprotein, Smooth muscle, βIII tubulin antibody overnight at 4°C. The following day, the sections were incubated with appropriate secondary antibodies for 30 min. The reaction was visualized using DAB (DAB-0031; Maixin-Bio) plus chromogen. Specimens were examined using a BX50 microscope (Olympus). For serum controls, 1% BSA in PBS was used in place of the primary antibody as a negative control.
